# Establishment and Expansion of *Harmonia axyridis* Pallas (Coleoptera: Coccinellidae) in Urban Green Areas in the Iberian Peninsula: From 2015 to 2021

**DOI:** 10.3390/insects13080741

**Published:** 2022-08-17

**Authors:** Roberto Meseguer, Belén Lumbierres, Xavier Pons

**Affiliations:** 1 Department of Crop Protection and Forest Sciences, University of Lleida, 25198 Lleida, Spain; 2 Laboratori d’Agricultura i Sanitat Vegetal de Catalunya, Departament d’Acció Climàtica, Alimentació i Agenda Rural, Generalitat de Catalunya, 25198 Lleida, Spain

**Keywords:** aphidophagous ladybirds, *Adalia bipunctata*, *Oenopia conglobata*, *Liriodendron tulipifera*, *Tilia platyphyllos*, aphids, invasive alien species

## Abstract

**Simple Summary:**

*Harmonia axyridis* (Coleoptera: Coccinellidae) is a widespread invasive ladybird. In this study, we determine its occurrence and expansion in urban areas of the Iberian Peninsula after 2014. We also define the aphidophagous ladybird species complex in urban areas of the northeastern Iberian Peninsula and track any changes between 2015 and 2021. The expansion of the species mainly occurred in Catalonia (northeast Iberian Peninsula), where spring and summer populations on trees and shrubs and overwintering aggregations were recorded. The records of *H. axyridis* populations allow us to distinguish between two areas: one that has already been invaded and another that has not yet been invaded. The relative abundance of the ladybird species in each area is determined on a yearly basis. In the invaded area, *H. axyridis* became dominant two years after its detection, replacing *Adalia bipunctata*. This change in species prevalence is more pronounced in trees highly infested with aphids. In the not yet invaded area, changes in species dominance also occurred, and *A. bipunctata* replaced *Oenopia conglobata* from 2020 onwards. The yearly release of this ladybird in urban green areas for aphid control purposes could explain this shift. This study defines the current situation of *H. axyridis* in the Iberian Peninsula, and determines the main changes in ladybird species assemblages during the last few years.

**Abstract:**

In the Western Palearctic region, *Harmonia axyridis* (Coleoptera: Coccinellidae) is mainly established in urban areas. In this study, we update its occurrence in urban areas of the Iberian Peninsula and determine its expansion after 2014. Changes in the ladybird species assemblage are also evaluated. We compile information about the records of *H. axyridis* in Spain from 2015 to 2021. In addition, we sample different locations to determine the relative abundances of the species composing the aphidophagous ladybird complex. The expansion of *H. axyridis* mainly occurred in Catalonia (Iberian Peninsula), where it is possible to identify two clear areas: one that has already been invaded and another that has not yet been invaded. *Harmonia axyridis* became the dominant species in the invaded area two years after it was first identified. This dominance is clearly shown on *Liriodendron tulipifera* and *Tilia platyphyllos*, where it accounted for more than 75% of the total collected individuals in the last year of the study. In the not-yet invaded area, *Adalia bipunctata* overcame *Oenopia conglobata* and became the prevalent species from 2020 onwards, likely due to its regular releases for aphid biocontrol. This study reveals that changes in ladybird species assemblages may not only be caused by invasive species, but also by biological control practices.

## 1. Introduction

Aphids (Homoptera: Aphididae) are widespread herbivores on plants of many ecosystems, including crops, forests, and urban vegetation [1]. They have the capacity to rapidly increase population size due to a high reproductive rate and short developmental time [2]. Fortunately, they have a large number of natural enemies that can stop or slow their population growth [3].

Ladybirds (Coleoptera: Coccinellidae) are among the most well-known aphid predators [3,4] and are considered to play a significant role as aphid control agents, especially in conservation strategies [5]. Each continent has a specific fauna of Coccinellidae [6], but the introduction of exotic species for aphid control may produce changes in this complex. In America, several aphidophagous species native to Europe (for example, *Coccinella septempunctata* L., *Hippodamia variegata* Goeze) were introduced for aphid control purposes, and their negative impacts have been well established [5,7,8]. In Europe, *Hippodamia convergens* Guérin-Meneville was introduced from North America, but there are no reports on its establishment [6,9]. The harlequin ladybird, *Harmonia axyridis* (Pallas), which is an East-Palearctic species native to Japan, China, Korea, Mongolia, and Siberia [10], was introduced to both North America and Europe. It has become established in many countries and has a well-documented impact on native ladybirds. *Harmonia axyridis* is able to prey on other ladybird species, avoid natural enemies, and better use food resources than the resident indigenous species; as a consequence, following their introduction, there was a change in the relative abundance of the ladybird complex [10,11,12,13,14,15,16,17].

In Spain, this coccinellid was introduced in Almería (southeastern Spain) for aphid control in 1995 [18]. Some adults were observed in the Canary Islands in 2003 and 2004 [19], and one single specimen was found in a public garden in Bilbao (northern Spain) in 2007 [20]. Despite this, no evidence of population establishment occurred [13,21] until Carbonell and Sesma [22] reported the occurrence of isolated individuals in several localities of Catalonia (northeastern Spain), and one overwintering aggregation quite close to the French border in 2013. Pons et al. [23] updated the status of this ladybeetle species in Spain and characterized an overwintering aggregation. Since then, some punctual new records of the occurrence of *H. axyridis* in the Iberian Peninsula have been made by citizen observers [24]. Nevertheless, no regular data on its expansion exists, and important changes may have occurred which could have led to a change in the relative abundance of the indigenous ladybird species.

The aims of the present study are as follows: (1) to update the occurrence of *H. axyridis* in the Iberian Peninsula and to determine its expansion after 2014; (2) to define the ladybird species composition in urban areas of the northeastern Iberian Peninsula; and (3) to track changes in the ladybird species assemblages throughout the study period.

## 2. Material and Methods

Firstly, we collected the available records from the literature on *H. axyridis* in Spain between 2015 and 2021, checking scientific databases (Web Science and Scopus), Spanish technical crop protection journals (Phytoma España, Vida Rural), technical reports from Spanish Agricultural services (Departament d’Acció Climàtica, Alimentació i Medi Rural, Generalitat de Catalunya; Ministerio de Agricultura, Pesca y Alimentación, Gobierno de España), Spanish entomological webpages and Google. The occurrence of winter aggregations after 2013–2014 was also recorded. Advisers and citizen observers informed us about their presence inside houses and buildings and, whenever possible, we visited them and recorded the number of individuals and color morphs in the aggregation.

We also prospected several locations in the northeastern Iberian Peninsula for the presence of ladybird aphidophagous species. Because *H. axyridis* spreads more rapidly into areas with a high proportion of urban land cover [11,25] and its preferential food tends to reach high densities in the vegetation of urban green areas [4], we monitored these types of habitats one or more times per season. In each of these locations, we selected trees and shrubs that we knew were likely to be infested by aphids at the sampling time. Each pair location/plant is referred to as a “site”. Vegetation in a site was inspected visually for 1 to 3 h (adding the sampling time of each observer), according to the number of plants to be inspected. The number of adults and larvae of each aphidophagous coccinellid species was determined, according to the recorder’s experience. When clusters of newly hatched larvae were found, they were brought to the laboratory and reared until we were able to determine the species. Because the number of samplings varied throughout a season and among sites, the total number of individuals observed of each species was standardized by dividing it by the number of samplings performed. The number of sites and the sampled plants are shown in Table S1.

With this information, a map with the expansion of *H. axyridis* in the northeastern Iberian Peninsula was drawn, showing the areas with and without the presence of this ladybird species. In order to determine changes in the ladybird species assemblages in these areas, we calculated the relative abundance of each ladybird species yearly from 2015 to 2021.

In one location, we regularly monitored two ornamental tree species between 2019 and 2021: tulip tree (*Liriodendron tulipifera*) and linden (*Tilia platyphyllos*), and we recorded the occurrence of aphidophagous ladybird species. These two plant species were selected because both usually support high densities of aphids (*Illinoia liriodendri* (Monell) on tulip tree, and *Eucallipterus tiliae* L. on linden). Samplings were performed every week or 15 days during the aphid infestation period (April–July). The sampling plan consisted of visual observation for 30 s of the tree canopy at two heights, at the low and the medium parts. The low canopy observation was made directly from the ground. For the upper canopy observation, a ladder was used, with the observer at 2.5 m above the ground level. The numbers of sampled trees were 50 and 30 tulip trees and linden, respectively, corresponding to 25% of the total number of planted trees in each sampling place. The number of adults and larvae of each aphidophagous coccinellid species was recorded. When clusters of newly hatched larvae were found, they were brought to the laboratory and reared until their identification at species level was possible. The relative abundance of each species each year was calculated.

### Statistical Analysis

To compare differences between years in the relative proportions of the different coccinellid species, a chi-square test was carried out. Pairwise comparisons between years were then performed after applying the Bonferroni correction. When the number of individuals of one or several species was very low, they were grouped for analysis. All statistical analyses were performed using R version 4.0.3 (R Foundation for Statistical Computing, Vienna, Austria).

## 3. Results

The main species composing the aphidophagous ladybird complex in the urban green areas of the northeastern Iberian Peninsula were: *Adalia bipunctata* L., *Adalia decempunctata* L., *Coccinella septempunctata* L., *H. axyridis*, *Hippodamia veriegata* Goeze, *Oenopia conglobata* L., *Propylea quatuordecimpunctata* L., and *Scymnus* spp. The infrequent aphidophagous ladybirds recorded were: *Myrhra octodecimguttata* L., *Harmonia quadripunctata* (Pontoppidan), *Chilocorus bipustulatus* L., *Oenopia lyncea* Oliv., *Tytthaspis sedecimpunctata* L., *Coccinula quatuordecimpustulata* L., *Calvia quatuordecimguttata* L., *Exochomus* sp., *Platynaspis* sp., and *Hyperaspis* sp.

### 3.1. Occurrence of H. axyridis

**Figure 1:**
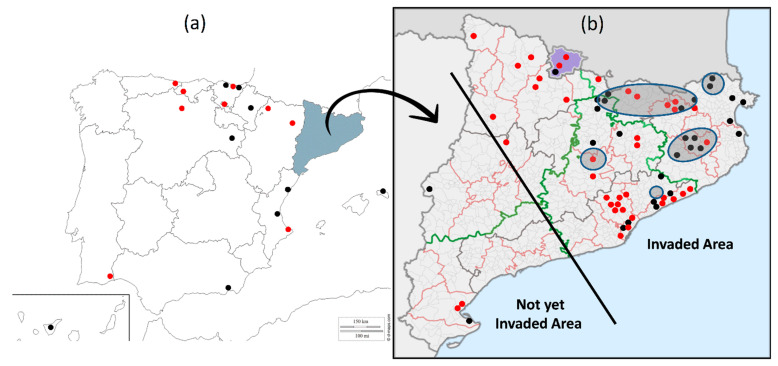
Localities of (**a**) the Iberian Peninsula and Canary and Balearic islands (except Catalonia) and (**b**) Catalonia and Andorra (in purple) where *H. axyridis* was recorded in spring or summer. Black and red points correspond to records before and after 2015, respectively. Grey ovals indicate the areas were overwintering aggregations have been found. The straight black line separates the updated hypothetical invaded area from the not yet invaded area within Catalonia.

By comparing the records reported before [23] and after 2014, this study revealed that *H. axyridis* has spread to more localities of the Iberian Peninsula (Figure 1a). However, most of these records correspond to isolated or small groups of adults that were photographed or observed by citizen scientists [24]. As far as we know, no records of winter aggregations have been reported in the Iberian Peninsula, except for those from Catalonia (Figure 1b).

The occurrence of *H. axyridis* in Catalonia is higher than in other parts of the Iberian Peninsula. Until now, spring and summer populations have been observed in more than 65 localities and 37 overwintering aggregations of between tens and hundreds of individuals have been reported in 5 different areas. The number of records after those reported in Pons et al. [23] has substantially increased (Figure 1b).

In Catalonia, the spread of *H. axyridis* has mainly occurred in the northern and eastern areas (Figure 1b). The isolated record in western Catalonia, belonging to the city of Lleida, corresponds to a solitary specimen that was found in 2014 inside a car coming from northeastern Catalonia. This individual was killed when detected [23]. No other specimens of *H. axyridis* have been found in the west, in a high number of urban areas (see Table 1a), on crops such as maize, alfalfa, cereals, orchards, etc., or in other habitats that are regularly sampled (authors unpublished). The records of the south of Catalonia come from localities of the Ebro delta area, where isolated individuals were photographed.

According to this information, we defined two areas in this region: one as the “invaded area” and the other as the “not yet invaded area” (Figure 1b).

### 3.2. Abundance of Ladybirds in the Not Yet Invaded and Invaded Areas

The not yet invaded area was dominated by *O. conglobata* until 2019, whereas *A. bipunctata* was the prevalent species in the last two years of the study (Table 1a). *Adalia bipunctata* was absent from this area until 2017, and its relative proportion increased over the years. The relative occurrence of *H. variegata* remained high every year and relatively stable. There were significant changes in the species occurrence over the study period (χ^2^ = 191.36; *p* < 0.0001; df: 36). Significant changes occurred in 2020 and 2021 (Table 1a and Table S2).

The invaded area was dominated by *A. bipunctata* until 2018. The appearance of *H. axyridis* was not recorded until 2017 and since this year there was a regular increase in its relative abundance in relation to the indigenous species (Table 1b). Significant changes in the relative abundance of ladybird species were also found over the study period in this area (χ^2^ = 771.15; *p* < 0.0001; df: 42). Yearly comparisons revealed that the *H. axyridis* arrival presupposed a significant shift in the coccinellid species complex composition in favor of the harlequin ladybird, with *A. bipunctata* being the most affected indigenous ladybird (Table 1a and Table S2).

**Table 1:** Yearly relative (%) and absolute (in parentheses) abundances of the different ladybird species in (**a**) the not yet invaded and (**b**) invaded area (from 2015 to 2021). The ladybird species have been abbreviated as: Ha (*H. axyridis*), A2 (*A. bipunctata*), Oc (*O. conglobata*), A10 (*A. decempunctata*), C7 (*C. septempunctata*), Hvar (*H. variegata*), P14 (*P. quatuordecimpunctata*), and Scy (*Scymnus* spp.). Years with different letters in the right-hand column are significantly different at *p* < 0.05. * See discussion for the reason of the low recorded value.

**(a)**										
		**Ladybird Species**								
**Year**	**Sample Sites**	**A2**		**Oc**	**A10**	**C7**	**Hvar**	**P14**	**Scy**	**Differences between Years**
2015	9	0 (0)		48 (26)	13 (7)	4 (2)	21 (11)	4 (2)	9 (5)	bc
2016	13	0 (0)		48 (40)	17 (14)	3 (2)	22 (18)	1 (1)	8 (7)	c
2017	16	4 (5)		40 (48)	12 (14)	2 (3)	28 (34)	5 (6)	9 (11)	bc
2018	16	11 (12)		37 (41)	12 (14)	2 (2)	27 (30)	3 (4)	6 (7)	bc
2019	13	15 (40)		36 (97)	8 (23)	1 (2)	34 (93)	2 (7)	4 (10)	b
2020	13	38 (117)		30 (93)	12 (36)	<1 (1)	14 (42)	1 (4)	5 (15)	a
2021	7	41 (33)		18 (14)	10 (8)	4 (3)	20 (16)	4 (3)	4 (3)	a
**(b)**										
		**Ladybird Species**								
**Year**	**Sample Sites**	**Ha**	**A2**	**Oc**	**A10**	**C7**	**Hvar**	**P14**	**Scy**	**Differences** **between Years**
2015	13	0 (0)	56 (178)	8 (25)	3 (10)	3 (8)	26 (84)	2 (7)	1 (4)	f
2016	9	0 (0)	53 (39)	5 (4)	9 (7)	6 (5)	29 (15)	1 (1)	3 (2)	ef
2017	10	6 (12)	46 (98)	8 (18)	12 (26)	5 (11)	15 (31)	4 (8)	5 (10)	de
2018	16	17 (29)	34 (57)	12 (20)	12 (20)	4 (7)	13 (23)	4 (6)	5 (9)	d
2019	19	52 (371)	24 (172)	6 (39)	3 (22)	2 (13)	13 (95)	<1 (1)	<1 (2)	c
2020	42	43 (559)	34 (442)	7 (85)	4 (56)	1 (11)	10 (128)	<1 (4)	1 (8)	b
2021	16	63 (279)	25 (109)	4 (17)	3 (13)	4 (18)	<1 (2) *	<1 (2)	<1 (2)	a

### 3.3. Changes in the Relative Abundance of the Aphidophagous Ladybird Species on L. tulipifera and T. platyphyllos

**Figure 2:**
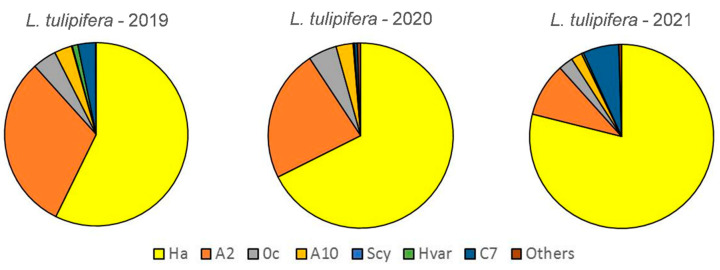
Relative abundance of the different ladybird species on *L. tulipifera* from 2019 to 2021.

*Harmonia axyridis* was the dominant species during the three years of sampling on *L. tulipifera*, followed by *A. bipunctata*. The former’s relative yearly abundance increased significantly over the period of 2019–2021 (χ^2^ = 426.46; *p* < 0.0001; df: 12) (Figure 2, Table S3).

**Figure 3:**
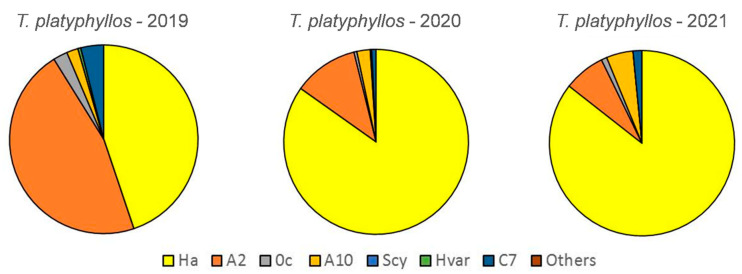
Relative abundance of the different ladybird species on *T. platyphyllos* from 2019 to 2021.

On *T. platyphyllos*, *A. bipunctata* was the prevalent species in 2019. However, a sharp increase in the abundance of *H. axyridis* relegated this species to second position from 2020. Changes in the species complex abundances were found over the study period (χ^2^ = 226.32; *p* < 0.0001; df: 10) (Figure 3). Yearly comparisons revealed significant differences between 2019 and 2020, but not between 2020 and 2021 (Table S3).

## 4. Discussion

The harlequin ladybird has an exceptional capacity to be successful in many new environments [26]. Its strong dispersal capacity makes this species capable of colonizing new areas with large enough aphid populations [10,27]. In the Western Palearctic region, *H. axyridis* is mainly established in urban areas [11,28,29], where aphid populations reach high densities on environmentally stressed plants (pollution, water, soil nutrient cycles) [25,30]. The focus on urban green spaces in our study allowed us to obtain the most informative data regarding the expansion of *H. axyridis* in the Iberian Peninsula since 2014, and the changes in the ladybird species assemblages after the establishment of this invasive species.

In the last two decades, much research has focused on the potential of the harlequin ladybird to invade new areas, the factors that may have led to such a successful expansion, and the consequences of this expansion. Some of these studies cast doubts on the invader’s ability to colonize Southern European regions, stressing that some biotic factors, such as the lower availability of food resources, may be playing a major role in preventing invasion by *H. axyridis* [31,32]. However, our study shows that the expansion of *H. axyridis* in the Iberian Peninsula [23] is in progress, especially in Catalonia (the northeast). Our findings regarding this expansion support predictions made by Poutsma et al. [33] and Amexia et al. [34] for the Mediterranean region and for the Iberian Peninsula, respectively. The expansion of *H. axyridis* in other southern European countries, like Bosnia-Herzegovina, Croatia, and Italy has been already reported [15,28,35,36].

Pons et al. [23] predicted a rapid spread of *H. axyridis* in crops due to the usual abundance of aphids during spring, as was reported by other European countries [37]. Amexia et al. [34] stated that the spread of *H. axyridis* in the northern Iberian Peninsula could be linked to vineyard areas, representing a risk for wine production as has already occurred in Canada [38], where damage to ripe bunches of grapes caused by this invasive ladybird species was reported during the harvest period. However, only a few adults of *H. axyridis* have been recorded in crop fields (authors unpublished), and surveys and interviews with vineyard farmers and wine producers in Catalonia have so far failed to detect the widespread presence of *H. axyridis*. Until now, its occurrence has mainly been restricted to urban areas. *Harmonia axyridis* prefers broadleaf arboreal host plants and concentrates in habitats with a high density of aphids [25]. The large aphid populations found on several trees in urban areas of Catalonia have likely facilitated the expansion of this invasive ladybird.

No regular reports of this species exist in southern areas of the Iberian Peninsula. The habitual high temperatures in these areas may be the key factor preventing its expansion. Recent studies have shown the detrimental effects of temperatures above 30 °C on the survival and fitness of this invasive species [39,40]. In addition, high temperatures can lead to a shortening of the period during which aphid populations are abundant enough to support the oviposition and development of coccinellids [32]. Nevertheless, new records from very hot climates [41] suggest that *H. axyridis* may be adaptable to extreme climates, so its potential spread to southern regions of the Iberian Peninsula should not be dismissed.

Pons et al. [23] stated that the impact of the establishment of the harlequin ladybird in the Iberian Peninsula would be similar to that already described in other European countries [42]. Some nuisance to humans in houses by overwintering aggregations have been reported in Spain, but no allergies or other skin reactions. Ecologically, the most concerning impact of the species’ expansion is the consequential changes in coccinellid assemblages [11,14,16,28,43,44,45,46,47].

Ladybirds are common aphid predators found in urban ecosystems [48,49]. Before the establishment of the harlequin ladybird, several aphidophagous species were prevalent in urban areas of Catalonia, including *O. conglobata*, *A. bipunctata*, *H. variegata*, *A. decempunctata*, and others [50,51]. This pattern is currently occurring in the not-yet invaded area, but it seems to have changed in the area where *H. axyridis* has spread and ladybird habitats overlap.

In the invaded area, *H. axyridis* has been established since 2017, and changes in the relative abundance of the species complex were recorded in 2019, 2020, and 2021. These changes were clearly observed on tulip trees and lindens. Kenis et al. [17] assessed the ecological risk posed by *H. axyridis* to 30 European native species and determined that *A. bipunctata*, *A. decempunctata*, and *O. conglobata* had the highest risk of displacement. Our results show that the most affected species may be *A. bipunctata*, because of the changes in population density observed on tulip trees and lindens. This is similar to the displacement of *A. bipunctata* already reported in other countries [17,30,47,52,53,54,55]. This can be explained due to the strong habitat overlap between *H. axyridis* and *A. bipunctata* [30], which may lead to strong competition between these two species. *Harmonia axyridis* is known to be the stronger competitor, due to its higher voracity, faster developmental time, and ability to gain more weight and to more quickly colonize resources [56,57]. Although several intraguild predation (IGP) studies have defined the two-spotted ladybird as the intraguild prey of *H. axyridis* [58,59], it seems that exploitative competition is the main cause of its decrease [47,60]. When *H. axyridis* reaches high numbers, *A. bipunctata* may be displaced to its ancestral habitats (i.e., natural or semi-natural habitats) [60]. The effect on the other species is not clear, and further studies are required to assess whether negative effects will arise from *H. axyridis* establishment. In the invaded area, the low relative abundance of *H. variegata* in 2021 was mainly due to the fact that its main host in urban areas, the oleander *Nerium oleander*, did not harbor large aphid populations, which may have caused the numbers recorded to be much lower than in previous years. An unexpected finding was the recording of a good number of *C. septempunctata* adults each year on tulip trees on just one of the summer sampling dates. The presence of only adults (no larvae were recorded at all) suggests that this ladybird uses tulip trees to obtain reserves before flying to other habitats or overwintering.

An interesting finding in the not-yet invaded area was the change in the most prevalent species. This was *O. conglobata* until 2019, but later *A. bipunctata* became the most abundant species. The reason for this shift is not clear. The advantages of one species may be due to its better performance in a concrete habitat, but no comparative studies between these two ladybirds have been done. However, some anthropogenic/human actions may enhance the success of one species. In many cities of the Iberian Peninsula, *A. bipunctata* has been widely released to control any aphid species (because it is the main aphidophagous ladybird that is commercially produced). We suggest that this could be the reason for the change in the relative abundance of the aphidophagous species in the not-yet invaded area. In areas where *H. axyridis* has already spread, releases of *A. bipunctata* are also undertaken annually. The results obtained in our study suggest that these regular releases have not had much of an effect on the dominance of *H. axyridis*.

## 5. Conclusions

The results of this study indicate the ongoing expansion of *H. axyridis* across the Iberian Peninsula. The expansion has thus far taken place mainly in Catalonia (northeast Iberian Peninsula), where two clear areas were defined: the invaded and the not yet invaded area. In the former, the presence of the exotic ladybird has been recorded since 2017, with it becoming the dominant species from 2019 onwards. The yearly increase of *H. axyridis* abundance was very clear on the ornamental trees *L. tulipifera* and *T. platyphyllos*, where it accounted for more than 75% of the total collected individuals in the last year of the study. In the not invaded area, *O. conglobata* was the prevalent species until 2020, when a sharp increase in the abundance of *A. bipunctata* relegated this species to second position. Such a shift could have had an anthropogenic origin, due to regular releases of *A. bipunctata* for aphid biocontrol. This study defines the current situation of *H. axyridis* in the Iberian Peninsula and points out that changes in the ladybird species assemblages may be caused not only by invasive species but also by biological control practices.

## Data Availability

The data presented in this study are available on request from the corresponding author.

## Supplementary Materials

The following supporting information can be downloaded at: https://www.mdpi.com/article/10.3390/insects13080741/s1, Table S1. Number of sampled urban areas and list of observed plants in both the not yet invaded and the invaded area. * Total number of sampled urban areas (not yet invaded area + invaded area); Table S2. Pairwise comparison between years to determine changes in the ladybird complex composition of the not yet invaded and the invaded area. Bold numbers indicate statistically significant differences; Table S3. Pairwise comparisons between years to determine changes in the ladybird complex composition of *Liriodendron tulipifera* and *Tilia platyphyllos*. Bold numbers indicate statistically significant differences.
