# Supplementary material for: Establishment and Expansion of Harmonia axyridis Pallas (Coleoptera: Coccinellidae) in Urban Green Areas in the Iberian Peninsula: From 2015 to 2021
Source: Insects. 2022 Aug 17;13(8):741. doi: 10.3390/insects13080741 (PMC9409829; doi:10.3390/insects13080741)
Supplement: Supplementary file 1 [file insects-13-00741-s001.zip › insects-1826538-supplementary.pdf]

Table S1. Number of sampled urban areas and list of observed plants in both the not yet invaded and the invaded area. *Total number of sampled urban areas (not yet invaded area + invaded area).

Year	# urban green areas	List of plants
2015	*22 (9 + 13)	*Celtis australis, Acer saccharinum, Acer campestre, Punica granatum, Populus* sp., *Liriodendron tulipifera, Punica granatum, Tilia* sp., *Lagerstroemia indica, Catalpa bignonoides, Quercus* sp., *Nerium oleander, Podranea ricasoliana, Viburnum lucidum, Phyllostachys* sp., *Arundo donax, Pittosporum tobira.*
2016	22 (13 + 9)	*Citrus aurantium, Acer saccharinum, Punica granatum, Populus* sp., *Prunus cerasifera-pisardii, Punica granatum, Tilia* sp., *Arbutus unedo, Hibiscus syriacus; Nerium oleander, Podranea ricasoliana, Viburnum lucidum, Phyllostachys* sp., *Pittosporum tobira, Urtica sp., Artemisia absinthium*
2017	26 (16 + 10)	*Citrus aurantium, Acer saccharinum, Punica granatum, Populus* sp., *Prunus cerasifera-pisardii, Punica granatum, Tilia* sp., *Arbutus unedo, Quercus* sp., *Hibiscus syriacus, Nerium oleander, Podranea ricasoliana, Viburnum lucidum, Phyllostachys* sp., *Pittosporum tobira, Urtica sp., Artemisia absinthium*
2018	32 (16 + 16)	*Citrus aurantium, Acer saccharinum, Punica granatum, Populus* sp., *Prunus cerasifera-pisardii, Punica granatum, Tilia* sp., *Arbutus unedo, Quercus* sp., *Hibiscus syriacus, Nerium oleander, Podranea ricasoliana, Viburnum lucidum, Phyllostachys* sp., *Pittosporum tobira, Urtica sp., Artemisia absinthiium*
2019	32 (13 + 19)	*Acer platanoides, Tilia* sp., *Populus* sp., *Arbutus unedo, Quercus* sp., *Quercus rubra, Liriodendron tulipifera, Carpinus sp., Robinia pseudoacacia, Juglans nigra, Nerium oleander, Podranea*

		ricasoliana, Viburnum lucidum, Phyllostachys sp., *Pittosporum tobira, Urtica sp., Spartium junceum*
2020	55 (13 + 42)	*Acer platanoides, Tilia* sp., *Populus* sp., *Arbutus unedo, Quercus* sp., *Quercus rubra, Liriodendron tulipifera, Carpinus sp., Robinia pseudoacacia, Prunus cerasifera, Prunus cerasifera-pisardii, Prunus avium, Alnus sp., Catalpa bignonoides, Juglans nigra, Budleia davidii, Nerium oleander, Podranea ricasoliana, Viburnum lucidum, Phyllostachys* sp., *Pittosporum tobira, Urtica sp., Spartium junceum, Viburnum lucidum, Arundo donax*
2021	23 (7 + 16)	*Acer platanoides, Tilia* sp., *Populus* sp., *Quercus* sp., *Quercus rubra, Liriodendron tulipifera, Prunus cerasifera-pisardii, Carpinus sp., Nerium oleander, Podranea ricasoliana, , Phyllostachys* sp., *Pittosporum tobira, Urtica sp., Spartium junceum*

Table S2. Pairwise comparison between years to determine changes in the ladybird complex composition of the not yet invaded and the invaded area. Bold numbers indicate statistically significant differences.

	Not yet invaded area			Invaded area		
	X^2-	df	p-value	X^2	df	p-value
2015 vs 2016	1.516	5	0.9112	9.148	6	0.1654
2015 vs 2017	4.3822	6	6.25E-01	51.848	7	**6.26E-09**
2015 vs 2018	8.4758	6	2.05E-01	96.389	7	**6.00E-18**
2015 vs 2019	19.428	6	0.003499	273.5	7	**2.73E-55**
2015 vs 2020	36.923	6	**1.82E-06**	246.64	7	**1.44E-49**
2015 vs 2021	34.41	6	**5.61E-06**	377.36	7	**1.70E-77**
2016 vs 2017	8.0089	6	0.2375	8.8761	7	0.2617
2016 vs 2018	12.89	6	0.04482	24.187	7	**0.001056**
2016 vs 2019	26.603	6	**1.72E-04**	86.647	7	**6.03E-16**
2016 vs 2020	47.757	6	**1.32E-08**	75.119	7	**1.36E-13**
2016 vs 2021	50.268	6	**4.15E-09**	165.61	7	**2.11E-32**
2017 vs 2018	4.4227	6	0.6197	16.403	7	0.02168
2017 vs 2019	16.93	6	9.55E-03	193.78	7	**2.38E-38**
2017 vs 2020	57.902	6	**1.20E-10**	176.34	7	**1.15E-34**
2017 vs 2021	46.699	6	**2.15E-08**	241.06	7	**2.21E-48**
2018 vs 2019	5.9596	6	4.28E-01	122.53	7	**2.27E-23**
2018 vs 2020	35.941	6	**2.83E-06**	112.85	7	**2.35E-21**
2018 vs 2021	26.933	6	**1.49E-04**	168.89	7	**4.30E-33**
2019 vs 2020	60.779	6	**3.13E-11**	35.253	7	**1.00E-05**
2019 vs 2021	36.01	6	**2.74E-06**	70.789	7	**1.02E-12**
2020 vs 2021	15.85	6	1.46E-02	105.86	7	**6.62E-20**

Table S3. Pairwise comparisons between years to determine changes in the ladybird complex composition of *Liriodendron tulipifera* and *Tilia platyphyllos*. Bold numbers indicate statistically significant differences.

	Liriodendron tulipifera			*Tilia plathyphyllos*		
	X^2	df	p-value	X^2	df	p-value
2019 vs 2020	84.783	6	**3.66E-16**	111.48	5	**2.00E-22**
2019 vs 2021	248.35	6	**9.26E-51**	198.69	5	**5.42E-41**
2020 vs 2021	330.34	6	**2.56E-68**	12.245	5	3.16E-02
